# Impact of marital status on renal cancer patient survival

**DOI:** 10.18632/oncotarget.19600

**Published:** 2017-07-26

**Authors:** Hongzhi Wang, Lu Wang, Ildar Kabirov, Li Peng, Guang Chen, Yinhui Yang, Zamyatnin Andrey A, Wanhai Xu

**Affiliations:** ^1^ Department of Urology, The 4th Affiliated Hospital of Harbin Medical University, Harbin, 150001, China; ^2^ Institute of Molecular Medicine, Sechenov First Moscow State Medical University, Moscow 119991, Russia; ^3^ Belozersky Institute of Physico-Chemical Biology, Lomonosov Moscow State University, Moscow 119992, Russia

**Keywords:** renal cancer, marital status, Surveillance, Epidemiology, and End Results, survival analysis, patient demographics

## Abstract

Marital status is an independent prognostic factor for various cancer types. The present study used the Surveillance, Epidemiology, and End Results (SEER) database of the National Cancer Institute (NCI) to analyze the impact of marital status on renal cancer patient survival outcomes. We identified a total of 62,405 eligible patients (23,800 women and 38,605 men). Overall 5-year renal cancer cause-specific survival (CSS) was 80.3% in the married group, 69.2% in the widowed group, 78.9% in the single group, and 76.5% in the divorced/separated group. The widowed patient group had the highest female/male ratio, more distant metastases, and fewer high-grade (III/IV) tumors. Most widowed patients (90.4%) were elderly (>60 years old). In our study, male renal cancer patients benefited more from marriage than females. We also found that white married patients had better survival outcomes than other white patient groups, but black unmarried and married patients exhibited similar survival outcomes. Our results show that, in general, unmarried patients have higher rates of cancer-specific mortality and highlight the importance of psychological intervention for cancer patients during treatment.

## INTRODUCTION

Renal cancer causes 140,000 deaths per year, and is the seventh most common cancer in the world [1]. In 2013, more than 350,000 people were diagnosed with renal cancer [1]. While most renal cancers are localized, low-grade tumors, nearly 17% of patients had distant metastases at the time of diagnosis [2]. Several factors, such as smoking tobacco [3], hypertension [4], obesity [5, 6], and red meat consumption [7], are associated with renal cancer progression. However, little is known about the roles of socioeconomic status and psychological supports, such as marital status, in renal cancer prognosis. Marital status is an independent parameter to predict survival outcome in various cancers [8–10], and married patients exhibit better survival outcomes than unmarried patients. Married status may be associated with improved social support, higher income, and healthier behaviors, which might improve cancer patient rehabilitation results [11–14]. To our knowledge, the effects of marital status on renal cancer patient survival have not yet been studied. Here, we collected data from the Surveillance, Epidemiology, and End Results (SEER) cancer-registry program, including individuals diagnosed with renal carcinoma between 2004 and 2013, and explored the impact of marital status on renal cancer patient cause-specific survival (CSS).

## RESULTS

### Patient characteristics

This study included 62,405 eligible renal cancer patients. Of these, 39627 (63.50%) were married, 6,674 (10.69%) were widowed, 9,346 (14.98%) were single, and 6,758 (10.83%) were divorced/separated (Table 1). The widowed patients group had the highest female/male ratio, more distant metastases, and fewer high-grade (III/IV) tumors (all P<0.001). Most widowed patients (90.4%) were elderly (>60 years old) (P<0.001).

**Table 1:** Patient baseline demographic and clinical characteristics

Characteristic	Total	Married	Widowed	Single	Divorced/separated	*P-*value
	(n=62405)	(n=39627) N(%)	(n=6674) N(%)	(n=9346) N(%)	(n=6758) N(%)	
**Sex**						<0.001
Male	38605	27104(68.4)	1824(27.3)	5941(63.6)	3736(55.3)	
Female	23800	12523(31.6)	4850(72.7)	3405(36.4)	3022(44.7)	
**Age**						<0.001
≤60	29301	18890(47.7)	639(9.6)	6169(66.0)	3603(53.3)	
>60	33104	20737(52.3)	6035(90.4)	3177(34.0)	3155(46.7)	
**Race**						<0.001
White	52479	34163(86.2)	5607(84)	7104(76)	5605(82.9)	
Black	5561	2489(6.3)	672(10.1)	1569(16.8)	831(12.3)	
AI	646	323(0.8)	69(1.0)	176(1.9)	78(1.2)	
API	3346	2399(6.1)	314(4.7)	426(4.6)	207(3.1)	
Unknown	373	253(0.6)	12(0.2)	71(0.8)	37(0.6)	
**Tumor size (cm)**						<0.001
≤7	44561	28486(71.9)	4721(70.7)	6499(69.5)	4855(71.8)	
>7	15632	9932(25.1)	1549(23.2)	2493(26.7)	1658(24.5)	
Unknown	2212	1209(3.1)	404(6.1)	354(3.8)	245(3.6)	
**Laterality**						<0.001
Left	30494	19271(48.6)	3298(49.4)	4569(48.9)	3356(49.7)	
Right	31265	20018(50.5)	3252(48.7)	4671(50)	3324(49.2)	
Bilateral	560	293(0.7)	107(1.6)	93(1)	67(1)	
Unspecified	86	45(0.1)	17(0.3)	13(0.1)	11(0.2)	
**SEER stage**						<0.001
Localized	42448	27238(68.7)	4232(63.4)	6384(68.3)	4594(68)	
Regional	9616	6332(16)	994(14.9)	1322(14.1)	968(14.3)	
Distant	9200	5512(13.9)	1143(17.1)	1470(15.7)	1075(15.9)	
Unknown	1141	545(1.4)	305(4.6)	170(1.8)	121(1.8)	
**Grade**						<0.001
I/II	31104	20315(51.3)	2827(42.4)	4579(49)	3383(50.1)	
III/IV	16256	10788(27.2)	1303(19.5)	2433(26)	1732(25.6)	
Unknown	15045	8524(21.5)	2544(38.1)	2334(25)	1643(24.3)	

### Effect of marital status on renal cancer patient CSS

**Figure 1:**
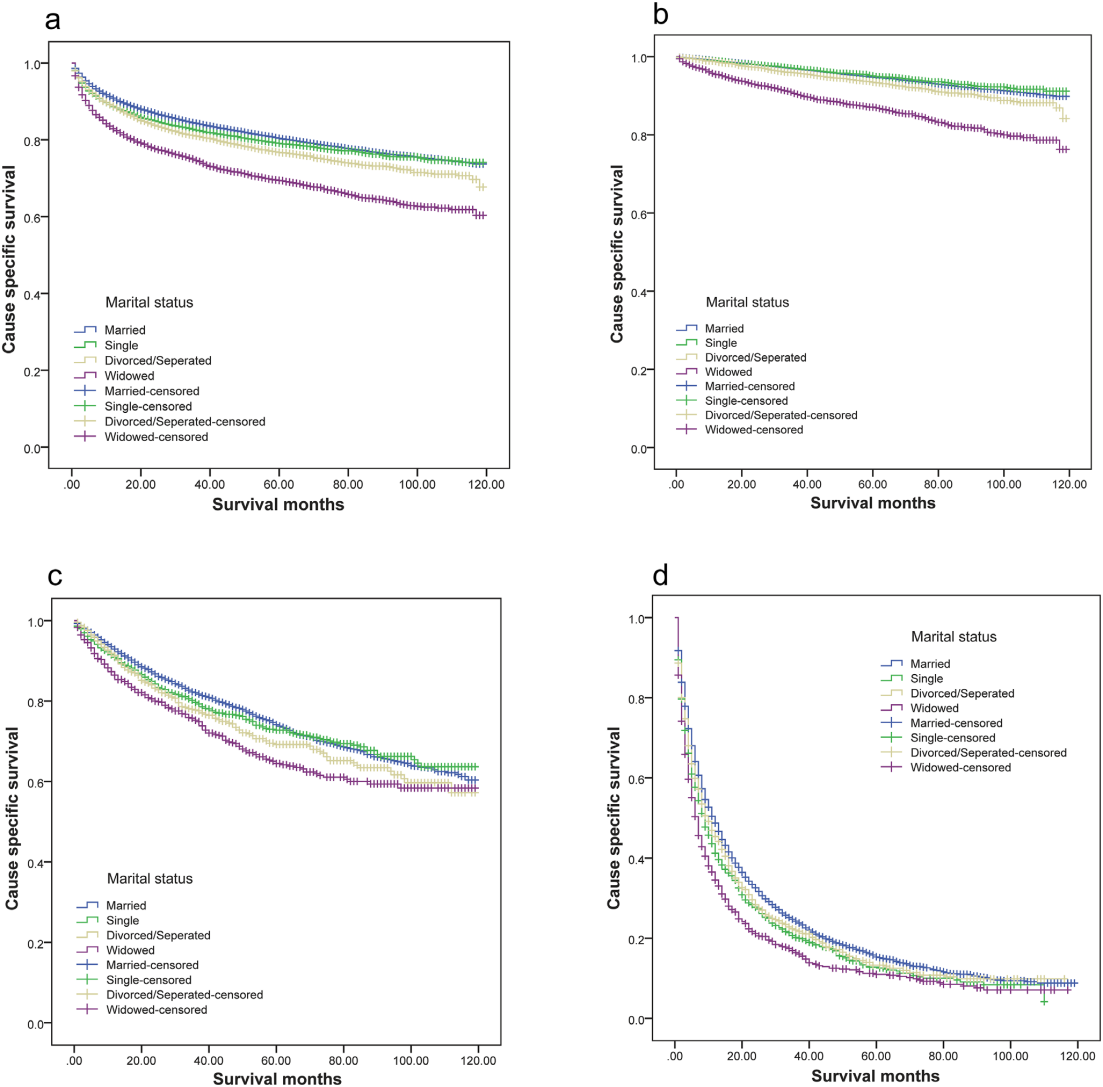
Survival curves of renal cancer patients according to marital status **(a)** All stage; χ^2^=441.757, P<0.001; **(b)** localized; χ^2^=407.581, P<0.001; **(c)** regional; χ^2^=33.455, P<0.001; **(d)** distant; χ^2^=80.016, P<0.001.

Overall 5-year renal cancer CSS was 80.3% in the married group, 69.2% in the widowed group, 78.9% in the single group and 76.5% in the divorced/separated group. (P<0.001, log rank test; Table 2, Figure 1A). Patient sex, age, race, tumor size, grade, laterality, and SEER stage were also identified as risk factors for cancer CSS.

**Table 2:** Univariate and multivariate survival analyses of the impact of marital status on renal cancer CSS

Variable	5-year CSS	Univariate analysis		Multivariate analysis	
		Log rank χ^2^ test	*P*-value	HR (95%CI)	*P*-value
**Sex**		76.381	<0.001		<0.001
Male	77.1%			Reference	
Female	80.7%			0.912(0.876–0.950)	
**Age**		739.793	<0.001		<0.001
≤60	83.5%			Reference	
>60	73.9%			1.233(1.185–1.282)	
**Race**		58.693	<0.001		
White	78.6%			Reference	
Black	76.6%			1.105(1.038–1.177)	0.002
AI	75.7%			0.949(0.800–1.124)	0.543
API	78.5%			1.046(0.963–1.136)	0.291
Unknow	93.4%			0.245(0.152–0.394)	<0.001
**Tumor size (cm)**		10876.88	<0.001		
≤7	88.7%			Reference	
>7	55.9%			2.477(2.379–2.580)	<0.001
Unknown	32.6%			1.350(1.253–1.456)	<0.001
**Laterality**		2523.04	<0.001		
Left	78.7%			Reference	
Right	79.5%			0.954(0.919–0.990)	0.013
Bilateral	17.0%			0.721(0.646–0.804)	<0.001
Unspecified	21.4%			0.646(0.497–0.840)	0.001
**SEER stage**		41800.0	<0.001		
Localized	93.9%			Reference	
Regional	72.3%			3.297(3.097–3.511)	<0.001
Distant	13.9%			19.840(18.740–21.004)	<0.001
Unknown	55.3%			1.957(1.703–2.249)	<0.001
**Grade**		9611.870	<0.001		
I	94.1%			Reference	
II	92.1%			1.259(1.122–1.413)	<0.001
III	75.7%			2.536(2.267–2.838)	<0.001
IV	47.1%			4.359(3.833–4.956)	<0.001
Unknown	56.5%			4.020(3.594–4.498)	<0.001
**Marital status**		441.757	<0.001		
Married	80.3%			Reference	
Widowed	69.2%			1.162(1.095–1.234)	<0.001
Single	78.9%			1.055(0.998–1.114)	<0.057
Divorced/Separated	76.5%			1.171(1.104–1.243)	<0.001

Multivariate analysis showed that marital status was also a prognostic factor. Widowed (hazard ratio [HR], 1.162; confidential interval [CI], 1.095-1.234) and divorced/separated (hazard ratio [HR], 1.171; confidential interval [CI], 1.104-1.243) patients had poorer outcomes than married patients, even after controlling for other risk factors.

The other covariates were also validated as independent factors in predicting renal cancer patient outcome. Female patients had better CSS than male patients (HR 0.912, 95% CI 0.876–0.950), while black patients had a higher mortality risk than white patients (HR 1.105, 95% CI 1.038–1.177). Patients with larger, advanced stage, or high-grade tumors, and elderly patients (>60 years of age) had higher mortality rates (Table 2).

### Subgroup analysis of the effects of marital status

We evaluated the impact of marital status on survival at each stage of renal cancer. Widowed and divorced/separated patients had worse survival rates compared with married patients in each tumor stage (Table 3, Figure 1). However, although single patients had a lower 5-year CSS than married patients with SEER distant stage tumors (12.5% vs 15.1%, P<0.001), multivariate analysis showed no difference between the two groups in localized (P=0.174), regional (P=0.410), and all tumor stages combined (P=0.057) (Table 3). Widowed patients had the lowest 5-year CSS. Compared to married patients, widowed patient 5-year CSS was 7.8% lower in localized stage (87.0% vs 94.8%, P<0.001), 9.8% lower in regional stage (64.2% vs 74.0%, P<0.001) and 4.3% lower in distant stage tumors (10.8% vs 15.1%, P<0.001). Compared with the married group, divorced/separated patient 5-year CSS was 1.4% lower in localized stage (93.4% vs 94.8%, P<0.001), 5% lower in regional stage (69% vs 74%, P<0.001) and 2% lower in distant stage tumors (13.1% vs 15.1%, P<0.001). Multivariate analysis also indicated that widowed and divorced/separated patients had worse survival outcomes (Table 3).

**Table 3:** Univariate and multivariate analyses of the impact of marital status on renal cancer CSS based on cancer stage

Variable	5-year CSS	Univariate analysis		Multivariate analysis	
		Log rank χ^2^ test	*P*-value	HR (95% CI)	*P*-value
**SEER Stage**					
**Localized**					
**Marital status**		407.581	<0.001		
Married	94.8%			Reference	
Widowed	87.0%			2.129(1.889–2.400)	<0.001
Single	95.1%			1.100(0.959–1.263)	0.174
Divorced/Separated	93.4%			1.399(1.221–1.603)	<0.001
**Regional**					
**Marital status**		33.455	<0.001		
Married	74.0%			Reference	
Widowed	64.2%			1.253(1.082–1.450)	0.003
Single	72.6%			1.0056(0.928–1.202)	0.410
Divorced/Separated	69.0%			1.192(1.036–1.372)	0.014
**Distant**					
**Marital status**		84.016	<0.001		
Married	15.1%			Reference	
Widowed	10.8%			1.158(1.067–1.257)	<0.001
Single	12.5%			1.131(1.055–1.213)	<0.001
Divorced/Separated	13.1%			1.081(1.002–1.167)	0.045

### Effect of marital status on renal cancer CSS by patient sex

**Figure 2:**
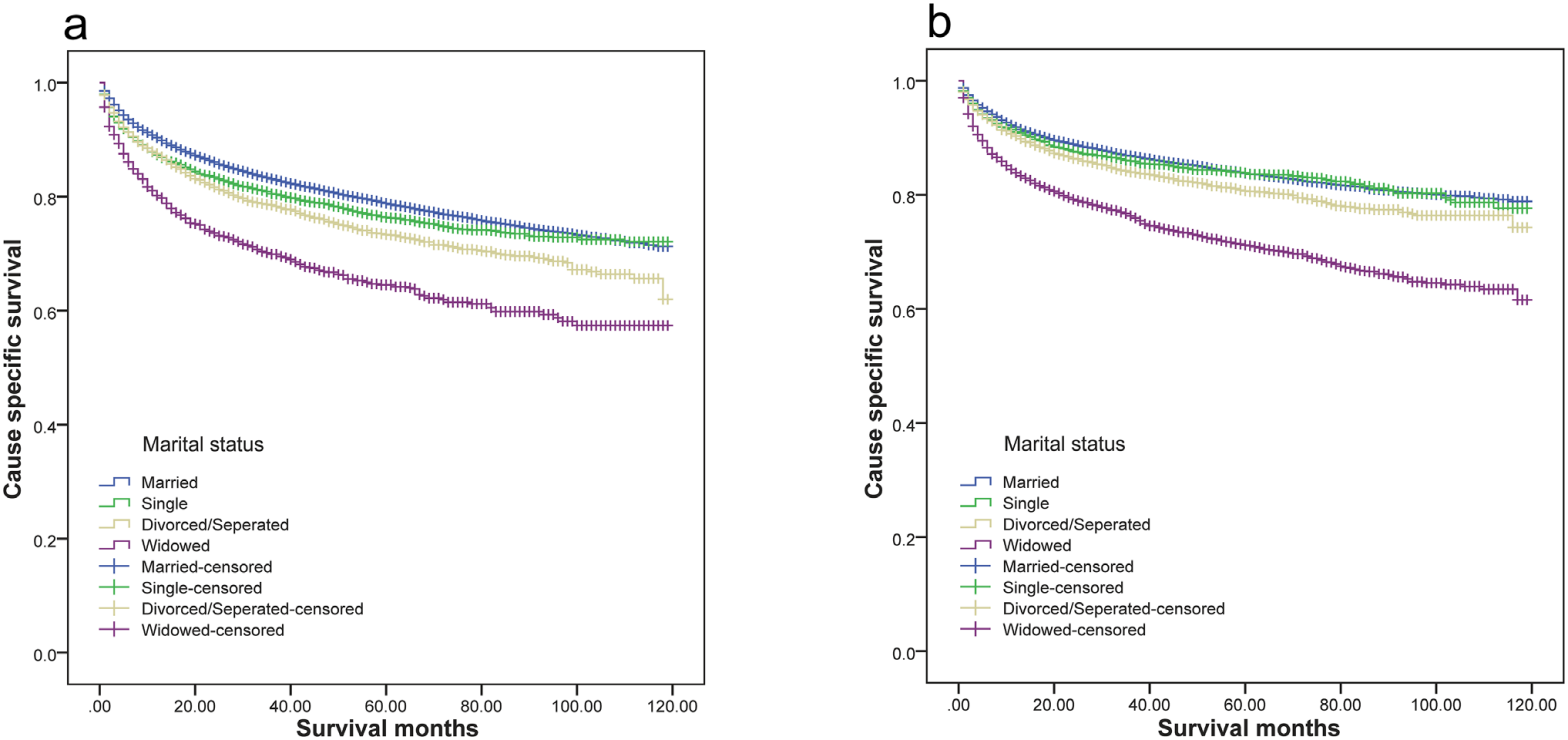
Survival curves of renal cancer patients according to marital status **(a)** Male: χ^2^=244.151, P<0.001; **(b)** female; χ^2^=366.889, P<0.001.

We analyzed the influence of marital status on male and female patient survival separately. Log rank χ^2^ test results indicated that marital status affected renal cancer CSS (P<0.001) in both men and women (Figure 2). Both female and male widowed patients had the lowest 5-year CSS. However, multivariate analysis showed that compared with married male patients, widowed (hazard ratio [HR], 1.282; confidential interval [CI], 1.171–1.404; P<0.001), single (hazard ratio [HR], 1.113; confidential interval [CI], 1.043–1.188; P=0.001) and divorced/separated male patients (hazard ratio [HR], 1.204; confidential interval [CI], 1.118–1.296; P<0.001) had poorer outcomes (Table 4). In female patients, only the divorced/separated group (hazard ratio [HR], 1.106; confidential interval [CI], 1.002–1.221; P=0.046) had poorer outcomes (Table 4).

**Table 4:** Univariate and multivariate analyses of the impact of marital status on renal cancer CSS based on patient sex

Variable	5-year CSS	Univariate analysis		Multivariate analysis	
		Log rank χ^2^ test	*P*-value	HR (95% CI)	*P*-value
**Sex**					
**Male**					
**Marital status**		244.151	<0.001		
Married	78.7%			Reference	
Widowed	64.3%			1.282(1.171–1.404)	<0.001
Single	76.2%			1.113(1.043–1.188)	0.001
Divorced/Separated	73.2%			1.204(1.118–1.296)	<0.001
**Female**					
**Marital status**		366.889	<0.001		
Married	83.7%			Reference	
Widowed	71.0%			1.065(0.983–1.154)	0.124
Single	83.8%			0.917(0.828–1.016)	0.098
Divorced/Separated	80.6%			1.106(1.002–1.221)	0.046

### Effect of marital status on renal cancer CSS by patient race

**Figure 3:**
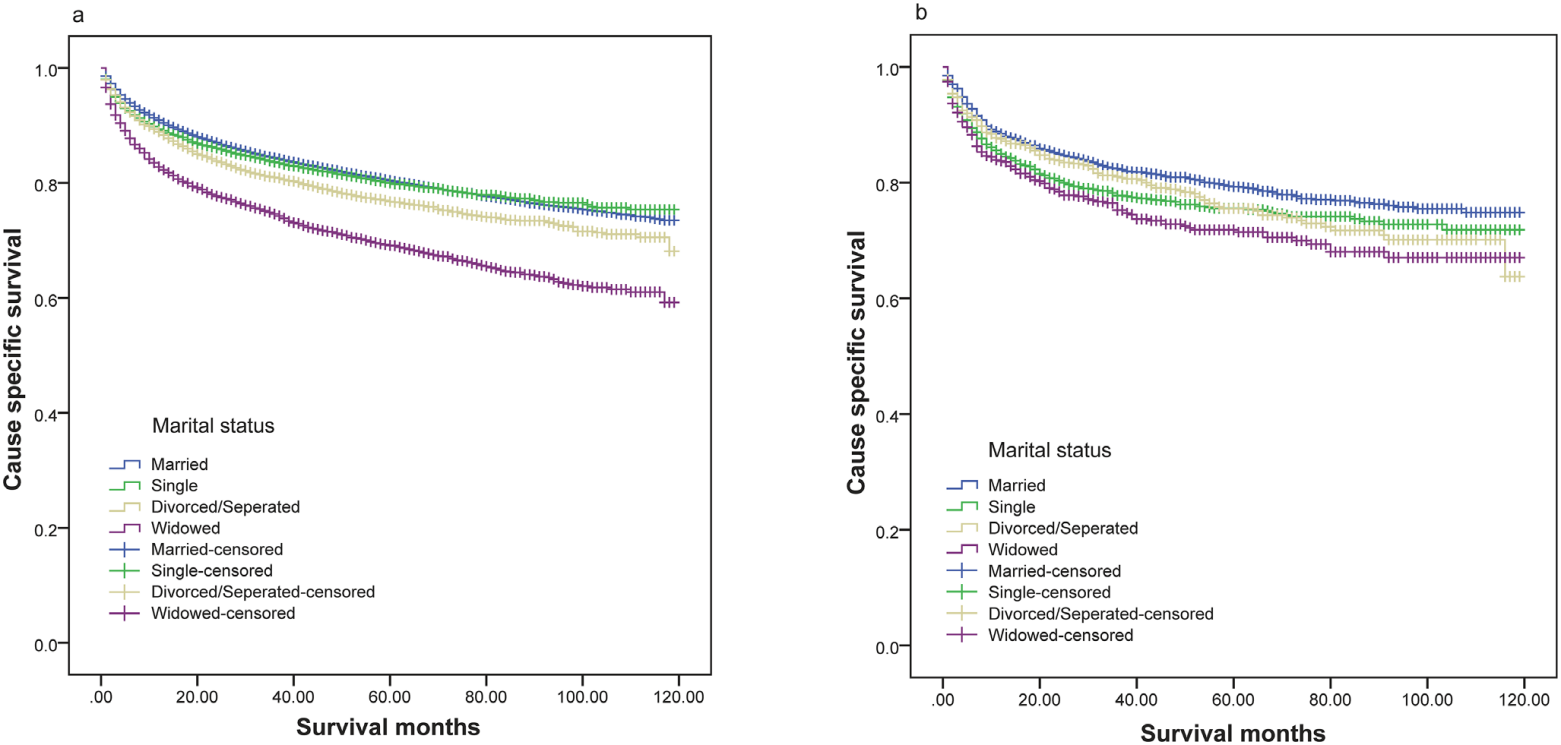
Survival curves of renal cancer patients according to marital status **(a)** White: χ^2^=394.370, P<0.001; **(b)** black; χ^2^=21.205, P<0.001.

Multivariate analysis results showed that black patients had worse survival outcomes than white patients, while other races had no survival differences compared with whites. Log rank χ2 test results showed that marital status affected renal cancer CSS (P<0.001) in both black and white patients (Figure 3). Similar to previous findings, white married patients had better survival outcomes than other white patient groups. However, black unmarried and married patients exhibited similar survival outcomes (Table 5).

**Table 5:** Univariate and multivariate analyses of the impacts of marital status on renal cancer CSS based on patient race

Variable	5-year CSS	Univariate analysis		Multivariate analysis	
		Log rank χ^2^ test	*P*-value	HR (95% CI)	*P*-value
**Race**					
**White**					
**Marital status**		394.370	<0.001		
Married	80.3%			Reference	
Widowed	68.9%			1.182(1.108–1.261)	<0.001
Single	79.8%			1.013(0.951–1.080)	0.679
Divorced/Separated	76.6%			1.158(1.085–1.235)	<0.001
**Black**					
**Marital status**		21.205	<0.001		
Married	79.2%			Reference	
Widowed	71.7%			1.038(0.824–1.280)	0.724
Single	75.4%			1.123(0.966–1.304)	0.130
Divorced/Separated	75.4%			1.149(0.959–1.378)	0.132

## DISCUSSION

The present study found that marital status is an independent prognostic factor for renal cancer, even after eliminating the influence of patient sex, age, race, tumor size, grade, laterality, and SEER stage. Widowed and separated/divorced patients had poorer survival rates compared with married patients. While a previous study suggested that male patients had better survival rates [15], female patients in our study had better 5-year CSS compared with male patients. As indicated by multivariate analysis, black patients had poorer outcomes than white patients. Patients with larger, more advanced stage, and higher-grade tumors had shorter survival times. Patients with bilateral tumors had the lowest 5-year CSS, possibly due to the presence of metastasis at diagnosis. Finally, patients with right-side renal tumors had slight better outcomes than those with left-side tumors. The reasons for this result need to be further investigated.

Our results also showed that male renal cancer patients benefited more from marriage than females. This trend was also observed in lung, colorectal, breast, pancreatic, prostate, liver/intrahepatic bile duct, non-Hodgkin lymphoma, head/neck, ovarian, and esophageal cancer patients in another SEER-based study [16]. A potential reason for this disparity is that male patients might receive more social supports from their relatives and friends [16]. However, a systematic review and meta-analysis found no differences with respect to the effects of marriage on male and female patient outcomes [12]. Race also influenced survival in patients with different marital statuses. Our results showed that marriage improved survival in white, but not black patients. Potential reasons for these findings must be clarified in future studies.

Although the association between marital status and cancer outcomes has been extensively investigated [8-10, 14, 15], the intrinsic mechanisms behind this association remain poorly understood. On explanation might be that better financial resources and health care are available to married patients, enabling earlier disease detection and more favorable treatment options [14]. However, a recent assessment of patient insurance and neighborhood socioeconomic statuses indicated that marriage-associated survival benefits may not mediated by better access to material resources [17]. Another possible explanation is that married patients have better access to social and psychological support [14]. Cancer patients usually experience distress and anxiety from the time of diagnosis [18, 19]. Without company and encouragement from a spouse, unmarried patients are more vulnerable to negative emotions. Evidence also suggests that psychological stress could promote tumor progression by disturbing normal immune and endocrine system functions [12, 14, 20]. Chronic psychological stress promotes cortisol secretion [21], which downregulates cortisol in white blood cells. This downregulation impairs cell responses to anti-inflammatory signals and induces excessive cytokine-mediated inflammatory processes [22], which are associated with cancer progression [23, 24]. Moreover, long term stress dysregulates immune function by altering the Type 1-Type 2 cytokine balance, inducing low-grade chronic inflammation, and inhibiting immune protective cell functions [25], all of which are associated with cancer metastasis [26–28]. Additionally, diurnal cortisol rhythms were associated with cancer patient survival [29, 30]. High quality psychological support from a spouse and perceived social support were associated increased natural killer cell activity [31]. Depression was also correlated with patient noncompliance with medical treatment recommendations [32].

This study had certain limitations. First, the SEER database only documented patient marital status at diagnosis. Patient marital statuses might have changed over the course of treatment, which could have influenced survival outcomes. Additionally, marriage quality might also impact spousal support levels, and this information was not included in the database. Second, the SEER database did not provide other important information, such as patient access to socioeconomic resources, which could influence the association between prognosis and marital status.

Despite these limitations, our study demonstrated a direct association between cancer patient marital status and prognosis based on a large and representative population. In summary, married patients had better survival rates than unmarried patients. We speculated that psychosocial and socioeconomic statuses might contribute to the better outcomes of married patients. This study highlights the importance of psychological intervention for cancer patients during treatment, especially for those who are unmarried.

## MATERIALS AND METHODS

### Data sources

All primary data were obtained from the SEER Program, including cancer incidence, stage, grade, therapy type(s), and population data, such as patient age, sex, race, and geographic region. The current dataset used for this study was based on Incidence-SEER 18 Regs Research Data + Hurricane Katrina Impacted Louisiana Cases, Nov 2015 Sub (1973–2013 varying).

### Patient selection and data extraction

We assessed renal cancer patients from the SEER database using SEER*Stat 8.3.2. Patients were selected according to the following criteria: (1) aged ≥18 years at diagnosis; (2) diagnosed between 2004 and 2013; (3) histological types were limited to clear cell adenocarcinoma and renal cell carcinoma (code 8310, 8312); (4) marital status was limited to married (including common law), divorced, separated, widowed, or single (never married). Patients with unknown cause of death, unknown survival time (months), or incomplete date information were excluded. Gender, age, race, marital status, grade, tumor size, laterality, SEER stage, cause of death, survival time, and survival months were extracted from the SEER database for each patient. This study was approved by the ethics committee of the Fourth Affiliated Hospital of Harbin Medical University (Harbin, China).

### Statistical analysis

Data were analyzed based on patient age, gender, race, marital status, tumor size, tumor grade, laterality, and SEER stage. Patients were divided into two age groups: ≤60 and >60. Race classifications included white, black, American Indian/Alaska native, Asian or Pacific Islander, and unknown. Divorced or separated patients were classified together into the divorced/separated group.

Patient baseline characteristics were analyzed using the χ^2^ test. Patient survival rates were calculated using the Kaplan-Meier method. A multivariate Cox regression model was built to analyze survival outcome risk factors. The primary endpoint of this study was cancer cause-specific death. Death resulting from renal cancer was assessed via events, and deaths due to other causes was considered censored events. All statistical analyses were performed using SPSS for Windows, v22 (SPSS Inc, Chicago, IL, USA). P<0.05 (two-sided) was considered statistically significant.
